# Congenital vaginal obstruction in a female with Cornelia de Lange syndrome: A case report

**DOI:** 10.3389/fendo.2022.886235

**Published:** 2022-08-25

**Authors:** Yiding Shen, Dongyan Zhao, Long Sun, Xiuzhen Yang, Xiang Yan

**Affiliations:** ^1^ Department of Urology, The Children’s Hospital, Zhejiang University School of Medicine, National Clinical Research Center for Child Health, Hangzhou, China; ^2^ Department of Ultrasound, The Children’s Hospital, Zhejiang University School of Medicine, National Clinical Research Center for Child Health, Hangzhou, China

**Keywords:** Cornelia de Lange syndrome (CdLS), urinary tract infection (UTI), vesicoureteral reflux (VUR), vaginal obstruction, case report

## Abstract

Cornelia de Lange syndrome (CdLS) is a rare genetic disease involving multiorgan systems that varies in clinical manifestations. Female genital abnormalities in patients with CdLS are rarely reported, and current guidelines for CdLS contain little information related to female genital abnormalities. We report a case of classic CdLS with an *NIPBL* gene pathogenic variant in a 4.5-year-old girl who experienced recurrent urinary tract infections (UTIs) with vesical tenesmus. Urogenital physical and imaging examinations revealed external vaginal orifice obstruction and bilateral vesicoureteral reflux (VUR). Vaginal diaphragm-like tissue resection and vaginal orifice plasty were performed on this patient. The symptoms of urination disorders and recurrent UTIs, as well as VUR grading, improved after relieving the vaginal obstruction during the operation. For female CdLS patients, especially those with VUR, it is necessary to check for genital abnormalities and perform timely treatment, which is of great significance in improving urination disorder symptoms, reducing resistance during voiding, decreasing the occurrence of secondary VUR, and controlling recurrent UTIs.

## Introduction

Cornelia de Lange syndrome (CdLS) is a rare genetic disease involving multiorgan systems; the occurrence of which is closely associated with gene pathogenic variant in the cohesin protein complex. It is characterised by specific facial features, growth and developmental delay, behavioral-cognitive impairment, and limb malformations. Most CdLS patients have multisystem malformations that vary in clinical manifestations (1). Female genital abnormalities in CdLS patients are rarely reported, and current guidelines for CdLS contain little information related to female genital abnormalities. To the best of our knowledge, there are no reports of CdLS cases with congenital vaginal obstruction. Here, we report the first case of CdLS with congenital vaginal obstruction in a 4.5-year-old girl, as well as our experience in the diagnosis and treatment of this case.

## Case presentation

A 4.5-year-old girl with CdLS was admitted to the Department of Urology at our hospital complaining of recurrent urinary tract infections (UTIs) and vesical tenesmus for 7 months. Seven months prior to admission, the patient had a recurrent non-febrile UTI, which could be improved by oral antibiotics; however, relapse occurred easily. Her mother noticed that post-void dribbling and vesical tenesmus always existed after the child received automatic micturition.

This patient was considered to have CdLS during infancy because of her distinct facial characteristics, including thick eyebrows, depressed nasal bridge, anteverted nares, flattened philtrum, and thin upper lip. Suggestive characteristics include developmental delay, intellectual disability, postnatal growth retardation, and short fifth fingers. The patient is of Chinese, Han nationality. A 1.395-kb deletion (chr5:37063809-37065204) encompassing exons 46–47 was detected in the *NIPBL* gene of the patient using next-generation sequencing (NGS) and quantitative polymerase chain reaction (qPCR). This child was diagnosed with classic CdLS based on genetic detection results and clinical findings (2). Based on the severity scoring system in CdLS proposed by Kline et al. (1), the patient presented a moderate phenotype and scored 17 points: 3 points in birth weight, 3 points in age of sitting alone, 1 point in age of walking alone, 1 point in age of saying the first word, 1 point in upper limb deformities, 5 points in the number of other main organ malformations, and 3 points for hearing loss. No abnormality was found during pregnancy of the patient’s mother: vaginal delivery, G1P1, full-term, and birth weight at 2.85 kg. No abnormality was observed on antenatal examination.

Urogenital physical examination revealed the vulva, small labia majora, and a thick diaphragm-like tissue covering the external vaginal orifice and part of the external urethral orifice.

**Figure 1:**
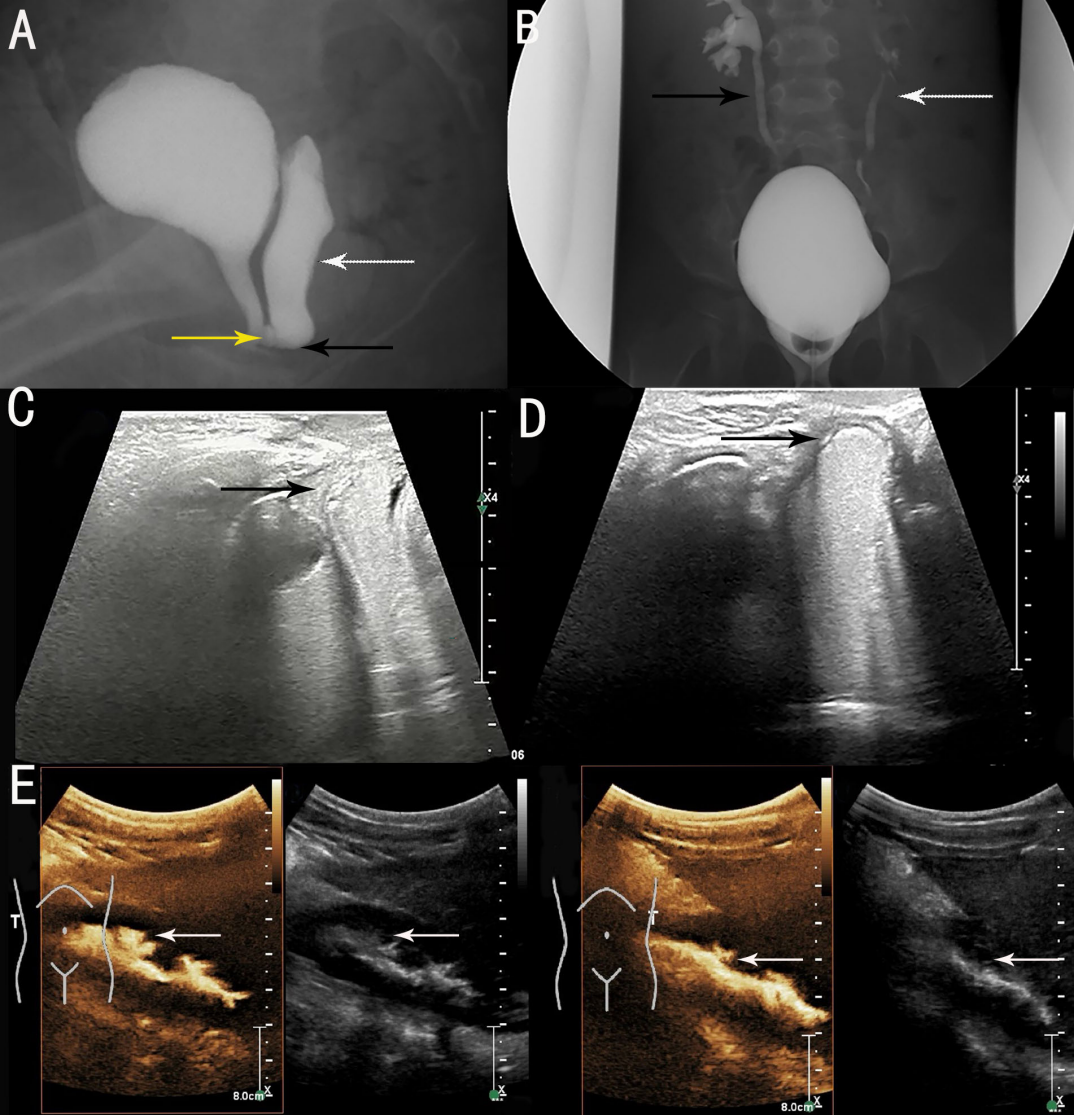
Preoperative imaging examinations. **(A)** voiding cystourethrography (VCUG) showed vaginal obstruction (black arrow), vaginal effusion (white arrow) and urethra-vaginal fistula (yellow arrow). **(B)** VCUG showed bilateral VUR (grade III on right side [black arrow] and grade II on left side [white arrow]). **(C)** contrast-enhanced voiding urosonography (ceVUS) revealed urethra-vaginal fistula (black arrow). **(D)** ceVUS revealed vaginal obstruction with vaginal effusion (black arrow). **(E)** ceVUS revealed bilateral VUR (grade III on right side and grade II on left side) (white arrows).

Genital ultrasonography showed: a) external vaginal orifice obstruction with vaginal effusion; b) urethra-vaginal fistula; and c) no obvious abnormality in the uterus or ovaries. Voiding cystourethrography (VCUG) revealed urethra-vaginal fistula, vaginal obstruction with vaginal effusion (**Figure 1A**), and bilateral vesicoureteral reflux (VUR) (grade III on the right side and grade II on the left side) (**Figure 1B**), and this was re-demonstrated on contrast-enhanced voiding urosonography (ceVUS) (**Figures 1C–E**). No significant renal scarring was noted on Tc-99m dimercaptosuccinic acid (DMSA) renal imaging.

**Figure 2:**
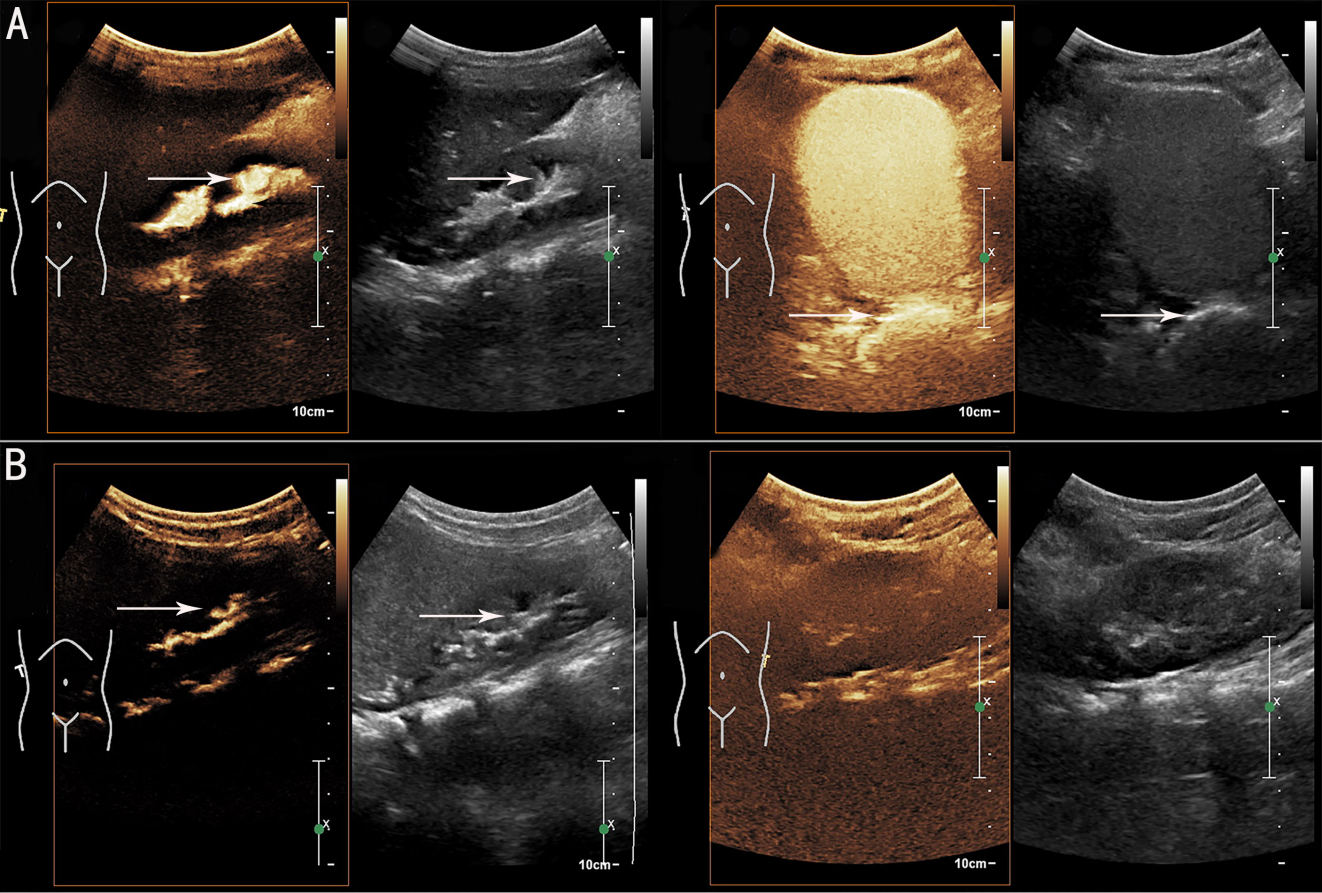
Postoperative contrast-enhanced voiding urosonography (ceVUS) results. **(A)** ceVUS showed bilateral vesicoureteral reflux (VUR) (grade III on right side and grade I on left side) (white arrows) 4 weeks postoperatively. **(B)** ceVUS revealed grade II VUR on right side (white arrows) and no VUR on left side 8 months after surgery.

Urethroscopy and colposcopy revealed that the external vaginal orifice was blocked by a diaphragm-like tissue, as well as a urethra-vaginal fistula with a diameter of approximately 3 mm. The patient was then treated with vaginal diaphragm-like tissue resection and vaginal orifice plasty. The patient had uneventful postoperative recovery. The urination disorder symptoms improved after surgery. Prophylactic antibiotics (nitrofurantoin) were used to treat the VUR. Repeated ceVUS was done 4 weeks postoperatively, which showed bilateral VUR (grade III on the right side and grade I on the left side) and no vaginal orifice obstruction with vaginal effusion (**Figure 2A**). Another repeated ceVUS showed grade II VUR on the right side, no VUR on the left side, and no vaginal orifice obstruction with vaginal effusion 8 months after surgery (**Figure 2B**). No recurrent UTI was observed during the 1-year postoperative follow-up (**Table 1**).

**Table 1:** Relevant clinical features, investigations, and treatment from the episode of care according to timeline.

Age(years)	Relevant clinical features, investigations and treatment
0.1	Diagnosed with classic CdLS followed by a regular follow-up; genetic test results showed a 1.395-kb deletion (chr5:37063809-37065204) encompassing exons 46–47 detected in the NIPBL gene
3.9	Routine urine test showed UTI and urinary urosonography showed no abnormality
3.9-4.4	Recurrent UTI, with an unsatisfactory therapeutic efficacy of antibiotics
4.4	VCUG revealed a urethra-vaginal fistula, vaginal obstruction with vaginal effusion, and bilateral VUR (grade III on the right side and grade II on the left side)
4.5	Admitted to the Department of Urology
	Preoperative ceVUS revealed a urethra-vaginal fistula, vaginal obstruction with vaginal effusion, and bilateral VUR (grade III on the right side and grade II on the left side)
	Operation: urethroscopy, colposcopy, vaginal diaphragm-like tissue resection and vaginal orifice plasty
	Prophylactic antibiotics (nitrofurantoin) were used postoperatively
4.6	Repeated ceVUS showed bilateral VUR (grade III on the right side and grade I on the left side) and no vaginal orifice obstruction with vaginal effusion
5.2	Another repeated ceVUS showed grade II VUR on the right side, no VUR on the left side, and no vaginal orifice obstruction with vaginal effusion
5.5	No recurrent UTI during the 1-year postoperative follow-up

CdLS, Cornelia de Lange syndrome; UTI, urinary tract infections; VCUG, voiding cystourethrography; VUR, vesicoureteral reflux; ceVUS, contrast-enhanced voiding urosonography.

## Discussion

According to the first international consensus in 2018, the diagnosis of CdLS is classified into classic, non-classic, or suspected types based on its main clinical characteristics and suggestive features. Further genetic detection (preferably NGS) is required for the diagnosis of non-classic or suspected CdLS, including the detection of at least five related genes (*NIPBL, SMC1A, SMC3, RAD21*, and *HDAC8*). The clinical phenotypes of CdLS patients vary significantly; thus, further scoring is required at 4 years of age to evaluate the severity (1). The patient was scored for distinct facial features during infancy and was diagnosed with classic CdLS based on genetic detection results. The patient was re-evaluated according to clinical phenotypes at 4 years of age and presented with a moderate phenotype.

At present, pathogenic variants of five or more of the above-mentioned genes have been found to be associated with the occurrence of CdLS (2). There is a correlation between genotypes and clinical phenotypes in patients with CdLS. A pathogenic variant in the *NIPBL* gene can be found in approximately 70% of cases (3), and several patients diagnosed with classic CdLS are found to carry mosaic pathogenic variant in the *NIBPL* gene (4). Patients with *NIPBL* gene pathogenic variant may have more severe clinical features compared with those carrying other gene pathogenic variants (5). *NIPBL*-encoded protein participates in the composition of the heterodimer complex, which is required for cohesin proteins to combine with chromosomes (6). Several studies have indicated that function deficiency variation in the *NIPBL* gene causes more serious clinical characteristics compared with misalignment variation in *NIPBL*, which is usually associated with more mild phenotypes (7, 8). *NIPBL* and *SMC1A* genes play critical roles in sister chromatid cohesin, chromosome cohesin, DNA repair, and expression regulation of developmental genes (9). However, several developmental defects in CdLS will arise if *NIPBL* or *SMC1A* encoded proteins are abnormally formed or truncated (10). Proteins encoded by *NIPBL* genes interact with cohesin proteins to perform most of the above-mentioned processes. The current patient showed multiple clinical manifestations, including genital abnormalities, possibly due to the fragment deletion (chr5:37063809-37065204) in the *NIPBL* gene.

To our knowledge, this is the first report of a female CdLS patient with congenital vaginal obstruction. CdLS with a fragment deletion pathogenic variant of the *NIPBL* gene often involves more multisystem malformations, including male genital abnormalities, such as cryptorchidism, micropenis, and hypospadias. The above-mentioned male genital abnormalities are usually found in childhood (2, 3). However, reports of CdLS patients with female genital abnormalities are few, and the associated clinical features may not arise until adolescence or adulthood. Boyle et al. (11) reported that CdLS patients had small labia majora and an abnormal uterus. Delayed or irregular menstruation during adolescence in CdLS patients has been reported by Kline et al. (12). In 2005, some researchers reported that an 18-year-old patient with CdLS was treated with hysterectomy due to hematometra caused by a uterine atony (13). Tate et al. have reported a female CdLS patient diagnosed with endometrial carcinoma in adulthood, followed by an operation (14). Congenital female vaginal obstruction commonly exists as a part of a complex syndrome, such as the Mayer–Rokitansky–Küster–Hauser (MRKH) syndrome, which could show different severities of vaginal dysplasia. It is reported that 10–15% of MRKH syndrome is caused by chromosomal abnormalities, the most common of which is the 1.2–1.9 Mb deletion at 17q12. However, the pathogenic role of these chromosomal abnormalities has not been fully clarified, and genotype–phenotype correlations are unclear (15). The McKusick–Kaufman syndrome, one of autosomal recessive disorders, is characterized by the combination of some congenital diseases, such as hydrometrocolpos, which is caused by vaginal agenesis, transverse vaginal septum, or vaginal atresia (16). However, as an independent developmental defect, congenital female vaginal obstruction has an extremely low incidence, and the specific molecular mechanism of vaginal development failure has not been clarified. Therefore, further exploration is required to study the molecular mechanism of the phenotype found in the present case. Congenital vaginal obstruction can occur in the general population in all age groups, whereas the treatment varies, whether in infancy, adolescence, or adulthood (17). In infancy and childhood, patients usually visit the hospital because of abdominal mass, sepsis, UTI, urination disorders, or other symptoms. The most common developmental malformations include imperforate hymen, transverse vaginal lower septum, urogenital sinus malformation, and cloacal malformation, and the treatments vary (18). An over-inflated vagina, followed by serious vaginal obstruction, can compress adjacent organs and cause conditions such as abdominal pain and urethral obstructive hydronephrosis, and symptoms of enuresis, urinary retention, urinary incontinence and recurrent UTI were reported in more than 50% of patients (19). Irregular menstruation, uterine abnormalities caused by outflow obstruction, and infertility could arise in adult patients with serious vaginal obstruction (20). In the present case, the patient mainly complained of recurrent UTIs with vesical tenesmus. These symptoms were associated with excessive vaginal expansion and compression of adjacent organs caused by vaginal obstruction, which was visible in her preoperative ultrasonography and VCUG examination. Probably due to the partial vaginal obstruction, there was no obvious clinical manifestation in infancy in this patient, while clinical symptoms such as urination disorders and recurrent UTIs gradually arose as the patient developed automatic micturition. Urination disorders in the child significantly improved after the operation, and the compression caused by vaginal obstruction was relieved.

At present, it has been reported that patients with CdLS often have VUR; therefore, attention should be paid to the urinary system for the diagnosis and treatment of CdLS (11). VUR refers to the retrograde flow of urine from the bladder into the upper urinary tract of the ureter and kidney. VUR is the main cause of UTI in children, with a higher incidence in females than in males (21). Its diagnosis and grading are mainly based on VCUG and ceVUS. Moreover, ceVUS has been widely used in the clinical diagnosis and grading of VUR owing to its accurate evaluation without radiation (22, 23). Persistent resistance during urination will reduce the therapeutic efficacy of VUR and may aggravate the condition. Improper treatment of VUR can cause recurrent UTIs, which result in renal scarring, and lead to the loss of renal function (21). In the present case, the patient had bilateral VUR (grade III on the right side and grade II on the left side) with recurrent UTI preoperatively, whereas the therapeutic efficacy of antibiotics was unsatisfactory. The patient’s VUR grading improved to grade III on the right side and grade I on the left side 4 weeks after the operation, during which vaginal obstruction was relieved. Furthermore, repeated ceVUS showed grade II VUR on the right side and no VUR on the left side 8 months postoperatively. No recurrent UTIs were reported at the 1-year postoperative follow-up. These changes indicate that relieving vaginal obstruction may directly reduce the grading of VUR and can significantly improve recurrent UTIs. This could effectively protect the kidney from the damage caused by bacterial vesicoureteral reflux.

However, there are potential limitations in this study. This patient did not go through another VCUG and DMSA after surgery, as the patient’s parents refused them for the concern of extra radiation exposure and the invasive procedure.

## Conclusion

For female CdLS patients, especially those with VUR, it is necessary to check for genital abnormalities and perform timely diagnostic procedures and adequate treatment, which will result in preventing urinary symptoms, reducing resistance during voiding, decreasing the occurrence of secondary VUR, and controlling recurrent UTIs.

## Patient perspective

The patient’s mother expressed her satisfaction at the current course of medical consultation. She was pleased that her daughter’s symptoms were relieved.

## Data availability statement

The datasets for this article are not publicly available due to concerns regarding participant/patient anonymity. Requests to access the datasets should be directed to the corresponding author.

## Ethics statement

Written informed consent was obtained from the individual(s), and minor(s)’ legal guardian/next of kin, for the publication of any potentially identifiable images or data included in this article.

## Author contributions

YS and DZ conceptualized and designed the study, analysed and interpreted the data, drafted the initial manuscript, and reviewed and revised the manuscript, equally contributing as principal authors. LS and XZY conceptualised and designed the study, analysed the data, drafted the manuscript, and critically revised the manuscript for important intellectual content. XY conceptualised and designed the study, interpreted the data, critically revised the manuscript for important intellectual content, edited the final manuscript, and provided supervision. All authors approved the final manuscript as submitted and agree to be accountable for all aspects of the work.

## Funding

This work was supported by Zhejiang Provincial Natural Science Foundation of China (grant LGF22H040006).

## Acknowledgments

We would like to thank Editage (www.editage.cn) for English language editing.

## Conflict of interest

The authors declare that the research was conducted in the absence of any commercial or financial relationships that could be construed as a potential conflict of interest.

## Publisher’s note

All claims expressed in this article are solely those of the authors and do not necessarily represent those of their affiliated organizations, or those of the publisher, the editors and the reviewers. Any product that may be evaluated in this article, or claim that may be made by its manufacturer, is not guaranteed or endorsed by the publisher.
